# Human papillomavirus serologic follow-up response and relationship to survival in head and neck cancer: a case-comparison study

**DOI:** 10.1186/1750-9378-6-9

**Published:** 2011-07-08

**Authors:** Linda M Rubenstein, Elaine M Smith, Michael Pawlita, Thomas H Haugen, Eva Hamšíková, Lubomir P Turek

**Affiliations:** 1 Department of Epidemiology College of Public Health, University of Iowa, Iowa City, IA 52242, USA; 2 Research Program infection and Cancer German Cancer Research Center (DKFZ), 69120 Heidelberg, Germany; 3 Veterans Affairs Medical Center and Department of Pathology, College of Medicine, University of Iowa, Iowa City, IA 52242, USA; 4 Department of Experimental Virology Institute of Hematology and Blood Transfusion, Prague, Czech Republic

**Keywords:** head and neck neoplasms, human papillomavirus, HPV-16 E6/E7

## Abstract

**Background:**

Human papillomavirus high risk (HPV-HR) type 16 is a significant risk factor for head and neck cancers (HNC) independent of tobacco and alcohol. The purpose of this study was to determine whether antibody levels to the HPV-16 oncoproteins E6 and E7 measured in sera collected at baseline (BL) prior to treatment and at two post-treatment follow-up (FU) visits were associated with HNC risk factors or prognosis.

**Methods:**

Presence of antibodies to HPV-16 E6 and E7 was evaluated in 109 newly diagnosed HNC cases with BL and FU blood samples, using the enzyme-linked immunosorbent assay (ELISA).

**Results:**

HPV-16 E6 and/or E7 seropositive HNC cases were associated with higher risk in younger patients (≤ 55 years), more sexual partners (≥ 10), oropharyngeal cancer, worse stage at diagnosis, poorer grade, and nodal involvement. Between BL and FU (median = 8.3 months), there were decreased antibody levels for seropositive E6 (73% vs. 27%, p = 0.02) and seropositive E7 patients (65% vs. 35%, p = 0.09) with 5% of BL E6 and 35% of BL E7 seropositive patients converting to negative status at FU. Overall mortality (OM) was significantly worse among BL E6 seronegative patients than among BL seropositive patients (40.2% vs.13.6%, p = 0.01). There were no disease specific (DS) deaths among BL E6 seropositive vs. 24% in BL E6 seronegative patients (p = 0.01). BL E7 seronegative patients also had higher mortality than BL seropositive patients (OM: 38.2% vs. 20.0%, p = 0.04; DS: 22.5% vs. 5.6%, p = 0.07).

**Conclusion:**

These findings are the first to follow post-treatment OD levels of HPV-16 E6 and E7 in HNC and suggest that these HPV antibodies may be potential prognostic markers of survival in HNC patients.

## Background

Human papillomavirus high risk (HPV-HR) types are causally related to cervical carcinomas [1] and a significant risk factor for approximately 26% of head and neck cancers (HNC) independent of tobacco and alcohol [2-7]. Survival and recurrence of HNC have not changed significantly over the past 30 years in the United States and Western Europe with recurrence remaining at ~30% [8]. There is recent evidence of better survival and lower recurrence among those detected with HPV-HR in tumors [9-12], suggesting that understanding HPV-HR infection is essential to understanding the prognosis of these tumors.

Several investigations have found concordance in HPV-16 positivity in tumor tissue and the presence of HPV specific antibodies in cervical cancer patients [13-15] and in HNC patients [3,16,7]. Antibodies to HPV-HR oncoproteins E6 and E7 are late markers for HPV-associated carcinoma with antibody prevalence increasing with clinical stage [17,18,15]. These tumors show constitutively high-level E6/E7 expression due to increased mRNA transcription and stability. Invasion of the E6/E7-overexpressed tumor cells beyond the mucosa brings them in tight contact with cells of the immune system and thus may lead to enhanced antigenic presentation and the induction of E6/E7 antibodies.

Several studies of cervical cancer have shown an association between decreased antibody level during pre-/post-treatment follow-up and better prognosis [19,13,14]. The purpose of this study was to examine changes in and impact of HPV antibody levels in HNC patients at baseline before treatment and at two follow-up periods after treatment to determine whether they are predictive of clinical outcomes.

## Materials and methods

### Patient population and study design

Newly diagnosed, primary HNC cases of the oral cavity (OC), oropharynx (OP), or larynx/hypopharynx (LH) were recruited between 2000-2005 at the University of Iowa Hospitals and Clinics, Department of Otolaryngology. They were administered a University approved Human Subjects Review consent form prior to interview and the study was approved by the institutional review boards at both the University of Iowa Hospitals and Clinics and the Iowa City Veterans Affairs Medical Center. All OC, OP, and LH sites as defined by the American Joint Commission on Cancer [20], excluding nasopharynx, were included. All histologies were included and subsequently combined because there were no significant differences in results between cases with squamous cell carcinoma (SCC, N = 102) and other histologic types (n = 7): 1 adenocarcinoma, 1 adenoid cystic carcinoma, 3 mucoepidermoid carcinoma, and 2 verrucous carcinomas. Cases included in this study (N = 109) had an initial baseline (BL) while 108 patients had a first follow-up (FU1) and 69 patients had a second follow-up (FU2) with blood samples available for evaluation of anti-HPV antibodies over a 3-24 month period.

Participants completed a risk factor questionnaire with information about demographics, sexual practices, tobacco and alcohol use, medical history, and history of HPV-related diseases and oral lesions. A medical form was completed using clinical medical records, supplemented by additional questions to the patient. Data included prior cancer history, cancer site, and treatments.

### Laboratory methods, serological assays, HPV detection and typing

The procedures performed on these clinical samples have been described previously [16]. Presence of IgG antibodies to antigens derived from HPV-specific proteins was evaluated using the enzyme-linked immunosorbent assay (ELISA) and have been described elsewhere [21]. Optical densities (OD) were recalculated into OD ratios dividing by the respective cutoff value. An OD ratio above 1.0 was considered positive. Changes in OD values between BL and FU were determined by comparing the last titration dilution from each visit before the OD ratio reached 1.0, the HPV detection cut off. DNA was extracted from deparaffinized tumor tissue sections with a QIAGEN DNA Tissue Kit (QIAGEN, Valencia, CA) and is described in more detail elsewhere [22]. All tumor tissue tested positive for the β-globin and PCR amplification of the cellular DNA verified adequate DNA [23]. DNA was PCR-amplified with MY09 and MY11 primers to detect HPV [24]. An aliquot of the PCR product of each HPV-negative specimen was hybridized by the dot blot method with ^32^P-labeled probes for detection of HPV DNA. Positive samples underwent hemi-nested PCR-amplification with MY09 and GP5+ primers [25]. DNA sequencing (Applied Biosystems-PE, Foster City, CA) was used to identify HPV types in comparison with GenBank sequences using the BLAST program [26].

### Statistical methods

The Wilcoxon rank sum test and a median test were used to compare quantitative variables between groups of patients. The chi-square or Fisher's exact test was used to compare categorical variables. Multiple logistic regression models [27], adjusting for continuous age, pack years and drinks per week, were used to estimate the odds ratios (ORs) and 95% confidence intervals (CI) for the association of baseline serology status with HNC risk factors and clinical characteristics of the tumors. For some variables with zero cells, ORs and CIs were generated with logit methods, adding 0.5 to zero cells and adjusting for categorical age (≤ 55 years vs. > 55 years), pack years (never, ≤ 30, > 30), and drinks per week (never, ≤ 21, > 21).

Each HPV serology test was evaluated at baseline (BL), defined as the time of diagnosis prior to any treatment, FU1 (n = 108, mean time to first follow-up = 7.0 months), FU2 (n = 69, mean time to second follow-up = 11.7 months), and FU (n = 109, mean time to the final follow-up (FU1 or FU2) = 10.1 months). Survival curves were generated using Kaplan-Meier methods, while Cox regression models were used to generate hazard ratios (HR) and 95% confidence intervals (CI), adjusted for continuous age and stage of disease (III/IV vs. I/II). Additional adjustment variables such as nodal involvement, tumor site, and grade could not be added to models because of the high correlation among these variables and with disease stage. Gender, pack-years and drinks per week were not associated with survival or recurrence and thus were not included in the models. All model variables met the proportion hazards assumption except for a disease specific model with E6 status because there were no deaths among the E6 seropositive patients. All reported p-values were two-sided, except where noted. One-sided p-values were based on hypotheses that the alternatives to a null finding would be decreased OD values (results supported by published cervical cancer research) [19,13,14]. Statistical analyses were performed using SAS version 9.2 [28].

## Results

### Patient characteristics, risk factors, and anti-HPV antibody response at baseline

Table 1 shows differences in patient risk factors and histopathologic characteristics by BL HPV serology status. Among the 109 cases, the majority was male (61%) and the average age was 59 years (range: 23-90 years). The overall antibody prevalence of HPV-16 E6 was 20%, HPV-16 E7 18%, and E6 and/or E7 24%. HPV-HR DNA (28 HPV-16 and 2 HPV-33)was detected in 28% of HNC tumors and the prevalence was significantly higher in the OP compared to the OC, 70% versus 15% (p < 0.0001). There were no HPV-HR tumors in the LH.

**Table 1:** Risk factors and histopathologic characteristics of HNC cases (N = 109)^1 ^by baseline HPV-16 E6/E7 status

Characteristics	HPV-16 E6^2^		Adjusted OR^5^	HPV-16 E7^3^		Adjusted OR^5^	HPV-16 E6 and/or E7^4^		Adjusted OR^5^
						
	Positive n (%)	Negative n (%)		Positive n (%)	Negative n (%)		Positive n (%)	Negative n (%)	
Gender									
Male	15 (68.2)	52 (59.8)	3.0 (0.9-9.4)	15 (75.0)	52 (58.4)	3.4 (1.01-11.5)	17 (65.4)	50 (60.2)	1.9 (0.7-5.2)
Female	7 (31.8)	35 (40.2)	1.0	5 (25.0)	37 (41.6)	1.0	9 (34.6)	33 (39.8)	1.0
Age Group									
≤ 55 years	14 (63.6)	30 (34.5)	2.7 (0.96-7.7)	12 (60.0)	32 (36.0)	2.7 (0.9-7.6)	15 (57.7)	29 (34.9)	2.5 (0.97-6.6)
> 55 years	8 (36.4)	57 (65.5)	1.0	8 (40.0)	57 (64.0)	1.0	11 (42.3)	54 (65.1)	1.0
Number of Partners									
0-2	7 (33.3)	35 (43.7)	1.0	6 (33.3)	36 (43.4)	1.0	9 (37.5)	33 (42.9)	1.0
3-9	5 (23.8)	23 (28.8)	1.4 (0.4-5.7)	4 (22.2)	24 (28.9)	1.3 (0.3-5.6)	5 (20.8)	23 (29.9)	1.0 (0.3-3.6)
≥ 10	9 (42.9)	22 (27.5)	6.9 (1.5-33.2)	8 (44.4)	23 (27.7)	5.6 (1.2-26.5)	10 (41.7)	21 (27.3)	4.5 (1.1-18.6)
Pack years									
Never	6 (27.3)	23 (26.4)	1.0	4 (20.0)	25 (28.1)	1.0	6 (23.1)	23 (27.7)	1.0
≤ 30	12 (54.5)	14 (16.1)	4.0 (1.1-14.1)	10 (50.0)	16 (18.0)	4.6 (1.2-18.3)	12 (46.2)	14 (16.9)	4.1 (1.2-14.3)
> 30	4 (18.2)	50 (57.5)	0.4 (0.1-1.8)	6 (30.0)	48 (53.9)	1.1 (0.2-4.7)	8 (30.8)	46 (55.4)	1.0 (0.3-3.5)
Drinks per Week									
Never	6 (27.3)	28 (32.2)	1.0	5 (25.0)	29 (32.6)	1.0	7 (26.9)	27 (32.5)	1.0
≤ 21	10 (45.5)	34 (39.1)	2.0 (0.6-6.6)	9 (45.0)	35 (39.3)	1.8 (0.5-6.1)	12 (46.2)	32 (38.6)	1.7 (0.6-5.2)
> 21	6 (27.3)	25 (28.7)	3.7 (0.8-18.1)	6 (30.0)	25 (28.1)	2.5 (0.6-11.3)	7 (26.9)	24 (28.9)	2.0 (0.5-7.8)
Tumor Site									
Oropharynx	20 (90.9)	7 (8.1)	51.9 (2.7-1007.0)^6^	16 (80.0)	11 (12.4)	4.1 (0.5-32.6)	20 (76.9)	7 (8.4)	7.9 (1.04-60.8)
Oral Cavity	2 (9.1)	71 (81.6)	0.7 (0.03-14.9)^6^	2 (10.0)	71 (79.8)	0.1 (0.01-0.7)^6^	4 (15.4)	69 (83.1)	0.2 (0.02-1.2)^6^
Larynx\hypopharynx	0 (0.0)	9 (10.3)	1.0	2 (10.0)	7 (7.9)	1.0	2 (7.7)	7 (8.4)	1.0
Oropharynx vs. Oral Cavity			19.7 (5.3-73.9)^7^			10.1 (2.9-35.8)^7^			24.2 (1.5-384.0)^7^
Stage									
I/II	2 (9.1)	38 (43.7)	1.0	1 (5.0)	39 (43.8)	1.0	3 (11.5)	37 (44.6)	1.0
III/IV	20 (90.9)	49 (56.3)	23.7 (4.2-133.6)	19 (95.0)	50 (56.2)	26.2 (3.1-221.8)	23 (88.5)	46 (55.4)	11.3 (2.8-45.8)
Tumor Size									
T0-T2	18 (81.8)	45 (52.9)	1.0	13 (65.0)	50 (57.5)	1.0	19 (73.1)	44 (54.3)	1.0
T3-T4	4 (18.2)	40 (47.1)	0.3 (0.1-1.04)	7 (35.0)	37 (42.5)	0.9 (0.3-2.6)	7 (26.9)	37 (45.7)	0.5 (0.2-1.4)
Grade									
Poor/Undifferentiated	11 (50.0)	14 (16.1)	5.5 (1.8-17.1)	9 (45.0)	16 (18.0)	3.7 (1.3-11.1)	12 (46.2)	13 (15.7)	4.8 (1.7-13.7)
Well/Moderate	11 (50.0)	73 (83.9)	1.0	11 (55.0)	73 (82.0)	1.0	14 (53.8)	70 (84.3)	1.0
Nodal Involvement									
Yes	20 (90.9)	31 (35.6)	37.3 (6.8-204.3)	17 (85.0)	34 (38.2)	11.3 (2.8-45.1)	21 (80.8)	30 (36.1)	9.4 (2.9-30.0)
No	2 (9.1)	56 (64.4)	1.0	3 (15.0)	55 (61.8)	1.0	5 (19.2)	53 (63.9)	1.0
HPV DNA									
High-Risk	20 (90.9)	10 (11.5)	22.0 (6.2-77.2)^7^	16 (80.0)	14 (15.7)	27.8 (7.2-107.8)	21 (80.8)	9 (10.8)	60.1 (13.9-259.4)
Type 16	19 (90.5)	9 (10.5)	25.1 (6.6,-95.2)^7^	16 (80.0)	12 (13.9)	32.5 (8.2-129.6)	20 (80.0)	8 (9.0)	58.0 (13.7-246.3)
Type 33	1 (0.4)	1 (1.0)	NE	0 (0.0)	2 (1.8)	NE	1 (0.8)	1 (1.8)	NE
Negative	2 (9.1)	77 (88.5)	1.0	4 (20.0)	75 (84.3)	1.0	5 (19.2)	74 (89.2)	1.0

^1^Number of patients; percentages (%) based on available data; ^2^Cases seropositive for HPV-16 E6 compared to cases seronegative for HPV-16 E6; ^3^Cases seropositive for HPV-16 E7 compared to cases seronegative for HPV-16 E7; ^4^Cases positive for E6 and/or E7 compared to cases negative for E6 and E7; ^5^Adjusted for continuous age, continuous pack years, and continuous drinks per week; ^6^Unadjusted logit estimator of OR; ^7^Logit estimator of OR and 95% CI., adjusted for categorical age, categorical pack years, and categorical drinks per week.

Males, those ≤ 55 of age and patients with ≥ 10 lifetime sexual partners were more likely to be E6, E7, or E6 and/or E7 seropositive. HNC patients with > 0/ ≤ 30 pack years had significantly higher ORs for seropositivity compared to never users, whereas patients who reported heavier alcohol drinking (> 21 drinks/week) had elevated ORs compared to never drinkers. OP tumors were associated with significantly elevated risks among those who were seropositive to BL E6, E7 and E6 and/or E7 when compared to OC or LH tumors. Stages III/IV vs. I/II, poorly or undifferentiated vs. moderate well differentiated tumors, and nodal involvement (yes vs. no) also were associated with elevated levels of BL E6 or E7 antibodies. Compared to HPV DNA negative tumor cases, HPV-HR tumor cases showed significantly higher risks for each HPV-16 specific BL E6 or E7 antibody. Of the 21 patients with HPV-HR tumors that were BL E6 and/or E7 seropositive, 20 tumors were HPV-16 and one was HPV-33. One HPV-33 DNA tumor positive case was BL E6 and/or E7 seronegative and 5 HPV DNA negative patients were BL seropositive.

Table 2 exhibits BL median HPV antibody OD ratios for risk factors and tumor characteristics at baseline among patients who were BL E6 or E7 seropositive. For each antibody group, there were no significant differences in median OD ratios by age and gender, although males displayed higher medians compared to females. There were non-significant higher median values for patients with three or more lifetime sexual partners versus 0-2 partners. There were no differences between never users and users of tobacco or alcohol within each E6 and E7 group or within E6 and/or E7.

**Table 2:** Baseline HPV-16 E6/E7 median OD^1 ^ratios^2 ^and ranges by risk factors, and histopathologic characteristics

Characteristics	HPV-16 E6		HPV-16 E7		HPV-16 E6 and/or E7	
	Positive (n = 22)		Positive (n = 20)		Positive (n = 26)	
	
	n (%)	Median (Range)	n (%)	Median (Range)	n (%)	Median (Range)
Gender						
Male	15 (22.4)	5.2 (1.8-11.3)	15 (22.4)	5.5 (1.3-37.2)	17 (25.4)	5.9 (1.3-37.2)
Female	7 (16.7)	4.7 (1.2-5.7)	5 (11.9)	2.1 (1.01-14.7)	9 (21.4)	4.7 (1.2-14.7)
Age Group						
≤ 55 years	14 (31.8)	5.3 (1.2-10.8)	12 (27.3)	4.9 (1.01-37.2)	15 (34.1)	5.5 (4.2-37.2)
> 55 years	8 (12.3)	5.0 (1.8-11.3)	8 (12.3)	5.7 (1.3-14.7)	11 (16.9)	5.8 (1.2-14.7)
Number of Partners						
0-2	7 (16.7)	4.7 (1.2-7.3)	6 (14.3)	1.8 (1.01-37.2)	9 (21.4)	4.6 (1.2-37.2)
3-9	5 (17.9)	5.9 (1.8-9.0)	4 (14.3)	4.9 (3.8-11.0)	5 (17.9)	7.3 (5.2-11.0)
≥ 10	9 (29.0)	5.0 (2.5-11.3)	8 (25.8)	5.5 (1.5-9.3)	10 (32.3)	5.6 (4.2-11.3)
Pack years						
Never	6 (20.7)	5.0 (1.2-9.0)	4 (13.8)	5.0 (3.8-9.3)	6 (20.7)	5.6 (1.2-9.3)
≤ 30	12 (46.2)	5.1 (2.5-10.8)	10 (38.5)	5.5 (1.01-37.2)	12 (46.2)	5.7 (4.3-37.2)
> 30	4 (7.4)	5.3 (1.8-11.3)	6 (11.1)	6.8 (1.3-14.7)	8 (14.8)	5.3 (1.3-14.7)
Drinks per Week						
Never	6 (17.6)	5.1 (1.2-7.8)	5 (14.7)	6.1 (1.3-9.3)	7 (20.6)	5.9 (1.2-9.3)
≤ 21	10 (22.7)	5.3 (2.5-10.8)	9 (20.5)	4.3 (1.01-37.2)	12 (27.3)	5.6 (2.1-37.2)
> 21	6 (19.4)	4.7 (1.8-11.3)	6 (19.4)	5.0 (1.5-11.0)	7 (22.6)	5.2 (4.2-11.3)
Tumor Site						
Oropharynx	20 (74.1)	5.1 (1.8-11.3)	16 (59.3)	5.7 (1.01-37.2)	20 (74.1)	5.9 (4.3-37.2)
Oral Cavity	2 (2.7)	2.6 (1.2-4.1)^3^	2 (2.7)	9.5 (4.2-14.7)^3^	4 (5.5)	4.2 (1.2-14.7)
Larynx\hypopharynx	0 (0.0)	-	2 (22.2)	1.7 (1.3-2.1)^3^	2 (22.2)	1.7 (1.3-2.1)^3^
Stage						
I/II	2 (5.0)	2.6 (1.2-4.1)^3^	1 (2.5)	1.3 (1.3-1.3)^3^	3 (7.5)	1.3 (1.2-4.1)*
III/IV	20 (29.0)	5.1 (1.8-11.3)	19 (27.5)	5.5 (1.01-37.2)	23 (33.3)	5.8 (2.1-37.2)*
Tumor Size						
T0-T2	18 (28.6)	4.9 (1.2-11.3)*	13 (20.6)	5.5 (1.01-37.2)	19 (30.2)	5.5 (1.2-37.2)
T3-T4	4 (9.1)	6.8 (5.2-10.8)*	7 (15.9)	5.5 (2.1-14.7)	7 (15.9)	5.9 (2.1-14.7)
Grade						
Poor/Undifferentiated	11 (44.0)	5.7 (4.2-11.3)	9 (36)	5.8 (1.01-37.2)	12 (48.0)	5.9 (1.3-37.2)
Well/Moderate	11 (13.1)	5.0 (1.2-10.8)	11 (13.1)	5.5 (1.6-14.7)	14 (16.7)	5.2 (1.2-14.7)
Nodal Involvement						
Yes	20 (39.2)	5.1 (1.8-11.3)	17 (33.3)	5.5 (1.01-37.2)	21 (41.2)	5.8 (4.2-37.2)*
No	2 (3.5)	2.6 (1.2-4.1)^3^	3 (5.2)	2.1 (1.3-14.7)	5 (8.6)	2.1 (1.2-14.7)*
HPV DNA						
High-Risk	20 (66.7)	5.0 (1.2-11.3)	16 (53.3)	5.7 (1.01-37.2)	21 (70.0)	5.7 (1.2-37.2)
Negative	2 (2.5)	7.1 (5.2-9.0)	4 (5.1)	3.0 (1.3-14.7)	5 (6.3)	5.2 (1.3-14.7)

^1^OD = optical density; ^2^All OD ratio tests were two-group comparisons: reference for partners was 0-2, reference for alcohol and tobacco was 'never', reference for tumor site was 'oropharynx'; ^3^Mean and range; *p-value for median difference between groups was ≤ 0.05.

The median HPV OD ratio for BL E6 and/or E7 antibodies (table 2) was elevated in the OP compared to the OC (5.9 vs. 4.2, p = 0.10). Compared to earlier stage disease, patients in stages III/IV had elevated medians within E6 and within E7 and had significantly higher medians for E6 and/or E7 (5.8 vs. 1.3, p = 0.008). Compared to patients with tumor size T1-T2, those with T3-T4 tumors had significantly higher OD medians for E6 antibodies (6.8 vs. 4.9 p = 0.05), but medians were similar for E7 antibodies and for the E6 and/or E7 group. There were no median OD differences by grade for E6, E7 and the E6 and/or E7 antibodies. The median OD in patients with nodal involvement compared to those without nodal involvement was elevated within E6 and within E7 and was significantly higher for E6 and/or E7 (5.8 vs. 2.1, p = 0.04).

### HPV antibody response at follow-up

Table 3 displays changes in the HPV OD ratios between BL and FU1, FU2 and final FU among patients seropositive at BL. Not all patients had a second FU (n = 40). At the last FU, over 72% of seropositive patients had a decrease in the anti-E6 ratio (p = 0.02) and 65% decreased in the anti-E7 ratio (p = 0.09). Although a majority of patients displayed a decreased OD ratio at the last FU, only 5% of E6 positive patient had converted from seropositive to seronegative (data not shown), while 35% of E7 positive patients had converted from seropositive to seronegative (*Kappas *for agreement = 0.8-0.97). The two seronegative patients who displayed a conversion to seropositivity between BL and FU1 were seronegative at FU2 (OD ratios: BL = 0.46, FU1 = 1.1, FU2 = 0.74 and BL = 0.54, FU1 = 2.0, FU2 = 0.46).

**Table 3:** Change^1 ^in seropositive OD ratios between baseline (BL) and follow-up (FU)^2 ^for BL seropositive cases

Change^1 ^in OD Ratio	HPV-16 E6 N = 22	HPV-16 E7 n = 20
	**n (%)**	**n (%)**
BL to FU1		
No decrease	8 (36.6)	10 (50.0)
Decrease	14 (63.4)	10 (50.0)
*p-value*^3^	0.10	0.50
BL to FU2		
No decrease	3 (25.0)	4 (36.4)
Decrease	9 (75.0)	7 (63.6)
*p-value*^3^	0.04	0.19
BL to last FU		
No decrease	6 (27.3)	7 (35.0)
Decrease	16 (72.7)	13 (65.0)
*p-value*^3^	0.02	0.09

^1^Based on ≥ 2-fold titration change using the last titration at each follow-up. ^2^FU1 = follow-up 1, FU2 = follow-up 2, FU = last follow-up, ^3^One-sided p-values for the binomial test that the proportion of patients who decreased was > 0.50.

The independent effects of treatment and E6/E7 antibody levels could not be assessed because treatment, tumor site, and seropositive status were highly correlated with one another. Treatment was significantly different by tumor site (p < 0.0001, data not shown). Over 95% of OC cases and 89% of LH cases had surgery, while only 33% of OP cases did. Chemotherapy was administered in 59% of OP patients, 1% of OC cases, and no LH cancer patients. In contrast, 89% of OP tumors had radiation compared to 47% of OC and 44% of LH tumors. However, 77% of the seropositive patients also had OP tumors, while only 15% of OC and 8% of LH tumors were seropositive.

Approximately 86% of all seropositive BL E6 patients and 85% of BL E7 had radiation therapy. Among all BL E6 seropositive patients whose OD ratio decreased, 94% had radiation therapy alone or with surgery and/or chemotherapy, whereas only 67% of patients with no changes in OD values had similar treatment regimens (p = 0.17, data not shown). Among all HPV seropositive BL E7 patients, 85% of those who decreased and 85% of those with no change, had radiation therapy. There was no association between those who decreased and those whose OD ratios remained unchanged among patients by surgical or chemotherapy status. There was no association between treatment type and conversion from HPV seropositive to seronegative at last FU.

**Figure 1:**
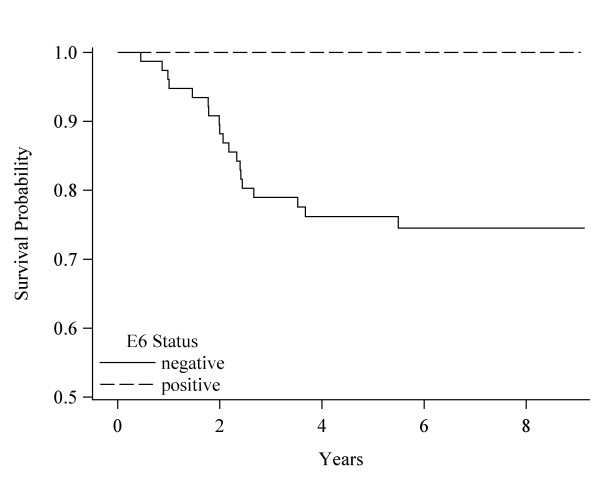
**Disease specific survival by HPV-16 E6 status**. Survival curves based on the Kaplan-Meier method. Significance was based on the Log-rank comparisons of positive vs. negative status, p = 0.02.

**Figure 2:**
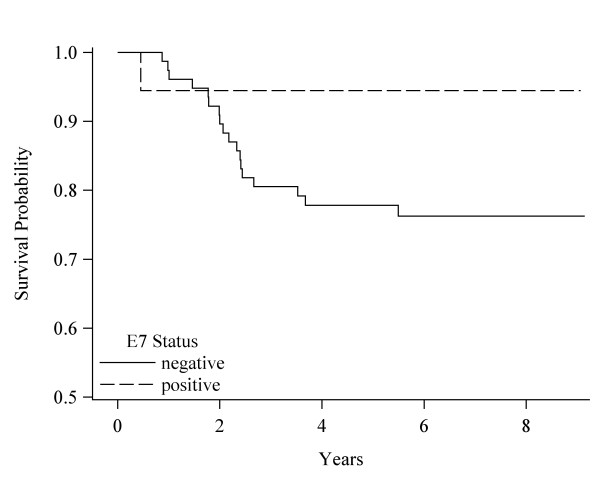
**Disease specific survival by HPV-16 E7 status**. Survival curves based on the Kaplan-Meier method. Significance was based on the Log-rank comparisons of positive vs. negative status, p = 0.11.

**Figure 3:**
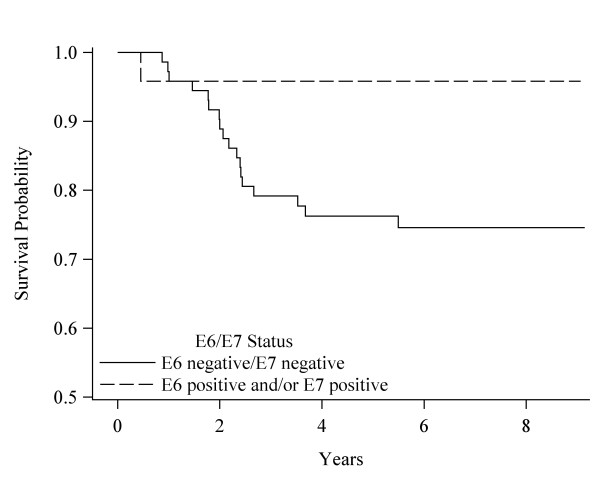
**Disease specific survival by HPV-16 E6 and/or E7 status**. Survival curves based on the Kaplan-Meier method. Significance was based on the Log-rank comparisons of positive vs. negative status, p = 0.04.

Table 4 displays the HRs (CIs) for OS, DS, and recurrence free survival between BL and last FU, comparing BL HPV seropositive to BL seronegative patients. The percent that died or had a recurrence as well as the change in OD ratios for these patients are also shown. There were too few deaths in BL seropositive patients to compare those with and without OD ratio reduction. OS included 109 patients, DS 98 patients, and recurrence free survival 101 patients. Among those who did not survive, 35% (n = 38) died from all causes combined, 19% (19) from HNC, and 19% (19) had a recurrence. Median follow-up time for OS was 6.2 years (range 0.5 to 9.1), for DS survival 6.4 years (range: 0.5 to 9.1), and for recurrence free survival 6.2 years (range: 0.3 to 9.1 years). For patients with a first recurrence, 84% occurred less than 20 months after diagnosis. For OS, 40% of the BL E6 seronegative vs. 14% of the BL E6 seropositive patients died (HR: 4.6); 38% of the BL E7 seronegative vs. 20% of the BL E7 seropositive did not survive (HR: 3.0); and 41% of the seronegative vs. 15% of the seropositive BL E6 and/or E7 died (HR: 3.8). Figures 1, 2, and 3 display the DS survival differences in seronegative and seropositive patients by BL E6, E7, and E6 and/or E7 status, respectively. DS death was 23%-24% among BL seronegative patients whereas there were no DS deaths among BL E6 positive patients (p = 0.01), one death among BL E7 positive patients (HR: 6.4) and one death among the BL E6 and/or E7 seropositive patients (HR: 8.4). There were no significant differences in recurrence between HPV BL seronegative and BL seropositive patients.

**Table 4:** Hazard Ratios by HPV-16 E6/E7 baseline status and change in OD ratio at last follow-up

Outcome/Antibody Status	HPV-16 E6			HPV-16 E7			HPV-16 E6 and/or E7		
	n (%)	HR (95% CI)^1^	p-value^2^	n (%)	HR (95% CI)^1^	p-value^2^	n (%)	HR (95% CI)^1^	p-value^2^
Overall Deaths			0.01			0.04			0.01
Negative	35^3 ^(40.2)	4.6 (1.4-15.3)		34^4 ^(38.2)	3.0 (1.03-8.5)		34^4 ^(41.0)	3.8 (1.3-11.0)	
Positive	3 (13.6)	1.0		4 (20.0)	1.0		4 (15.8)	1.0	
No OD decrease	0			0			0		
OD Decrease	3^5^			4^5^			4^5^		
									
Disease Specific Deaths			0.01^7^			0.07			0.04
Negative	19^6 ^(24.4)	NE^3^		18^8 ^(22.5)	6.4 (0.8-48.7)		18^8 ^(24.3)	8.4 (1.1-63.3)	
Positive	0 (0.0)	1.0		1 (5.6)	1.0		1 (4.2)	1.0	
No OD decrease	0			0			0		
OD Decrease	0			1^5^			1^5^		
									
Recurrence			0.35			0.36			0.90
Negative	17^8 ^(21.5)	2.1 (0.4-9.7)		17^4 ^(21.0)	2.0 (0.4-9.4)		15^9 ^(20.0)	1.1 (0.3-3.5)	
Positive	2 (9.1)	1.0		2 (10.0)	1.0		4 (15.4)	1.0	
No OD decrease	1^5^			0			2^5^		
OD Decrease	1			2^10^			2^10^		
									

^1^Adjusted for continuous age and stage; ^2^Adjusted p-value from Cox Regression models; ^3^Tumor status: 3 HPV-16, 1 HPV-33, 27 HPV-; ^4^Tumor status: 2 HPV-16, 1 HPV-33, 27 HPV-; ^5^Tumor status: all HPV-16; ^6^Tumor status: 2 HPV-16, 18 HPV-; ^7^Not estimable, Kaplan-Meier log-rank p = 0.01; ^8^Tumor status: 1 HPV-16, 1 HPV-33, 14 HPV-; ^9^Tumor status: 1 HPV-33, 13 HPV-; ^10^Tumor status: 1 HPV-16, 1 HPV-.

When compared by HPV-HR tumor positive vs. negative status (data not shown), the mortality rate for OS were 27% vs. 38% (HR: 2.1, 0.9-4.6, p = 0.07), for DS deaths, 12% vs. 22% (HR: 2.6, 0.7-9.0, p = 0.14) and for recurrence, 14% vs. 21% (HR: 1.5, 0.5-4.6, p = 0.49). The patient who was BL E6 and/or E7 seronegative and HPV-33 tumor positive died from HNC (survival time = 1.8 years) and also had a recurrence (time to recurrence = 1.2 years). The patient who was BL E6 and/or E7 positive and HPV-33 tumor positive was alive and disease free at last contact (follow-up time = 6.3 years). Of the 5 E6 and/or E7 seropositive patients who were HPV tumor negative, none had died (mean (SE) follow-up time = 5.4 (0.7) years, range 3.1-7.4 years) and one had a recurrence at 20 months.

## Discussion

This is the first study to describe the association between risk factors, pathological characteristics, and HNC survival with specific anti-HPV antibody levels at baseline and follow-up after treatment as has been done in cervical cancer studies [13,29,30,14]. The purpose for evaluating OD values at FUP in association with treatment was to determine whether treatment had an effect on OD levels. In cervical cancer, following OD levels over time after treatment has shown both an increase in survival and lower recurrence in those whose OD levels decreased. In this study, we found that median OD values for HPV-16 E6 and/or E7 antibodies at baseline were not consistently associated with risk factors for HNC. In contrast, between BL and the last FU, there was a statistically significant reduction in E6 antibodies and a non-significant decrease in E7 antibodies. In addition, overall and disease specific survival were better among the BL E6 and/or E7 seropositive patients compared to seronegative patients, reflecting significantly lower mortality in patients with BL E6 antibodies and also (non-statistically significant) lower mortality in patients with BL E7 antibodies.

Although there are no large studies of HPV antibody levels in HNC prior to and following treatment, studies of cervical cancer have examined pre/post-treatment titers for associations with risk factors, tumor characteristics, and clinical outcomes [13,29,30,14] and may provide a model for comparison. Cervical cancer research has suggested that decreases in E6 or E7 antibody levels between pre-and post-treatment were associated with continuing remission [13,29,30,14]. In contrast, persistent or increasing antibody levels showed evidence for continuing or recurrent disease [29,30,14]. Hamšíková et al. [14] found decreases in E7 antibody levels after radiation treatment of advanced cervical cancer cases. Fisher et al. [30] observed significant decreases in both HPV-16 E6 and E7 levels after radiation treatment. Baay et al. [19,13] reported similar findings but did not distinguish changes by treatment. Di Lonardo et al. [29] found no decrease in serum E7 levels for cervical cancer patients in stages IIB and IIIB three months after chemotherapy, but did observe a significant E7 OD decrease in 90% of the women after surgery regardless of tumor stage and prior chemotherapy.

In this HNC study, 73% of BL E6 positive and 65% of BL E7 positive patients exhibited decreases in OD levels with 85%-94% of those with a decrease also receiving radiation treatment alone or in combination with other therapies. These treatment rates reflected the high correlation between seropositivity and OP cancer and seronegativity and OC cancer. Eighty percent of stage III/IV E6 positive patients and 63% of stage III/IV E7 patients also exhibited decreases in their OD ratios. The decline in E6 and/or E7 antibodies after tumor removal or radiation suggest that these viral oncoproteins play a role in tumor genesis and growth in patients infected with HPV-HR. Identifying the metabolic pathway of these oncoproteins may indicate new targets for more effective therapies.

E6 and/or E7 prevalence rates at baseline and at the last FU in our HNC cases were difficult to compare to the prevalence rates in the study by Zumbach et al. [7] because the distribution of tumors was very different by site and it is well-established that OP cases are more likely to be HPV positive [8]. Over 65% of the Iowa tumors were from the OC, 25% from the OP, and 8% from the LH. In the German study, the proportions were approximately 4%, 37%, and 49%, respectively. In addition, Zumbach compared two different groups of patients, one with sera collected around the time of diagnosis and another with sera collected 6-26 month after diagnosis and treatment.

## Conclusions

Serology tests of HPV titer levels performed at pre-and post-treatment provide useful markers of clinical outcomes of cervical cancer. HNC serologic assessment of HPV E6 and E7 antibody changes may be a useful clinical method for monitoring patient treatment response and early warning of recurrence. Further investigation of changes in OD levels in HPV-associated head and neck tumors needs to be performed

## List of Abbreviations

BL: Baseline visit; CI: confidence intervals; DS: disease specific; ELISA: enzyme-linked immunosorbent assay; FU: Follow-up; FU1: Follow-up visit 1; FU2: Follow-up visit 2; HPV-HR: human papillomavirus high-risk; HR: hazard ratios; LH: larynx/hypopharynx; OC: oral cavity; OD: optical density; OM: overall mortality; OP: oropharynx; OR: odds ratios; OS: overall survival; SCC: squamous cell carcinoma.

## Competing interests

The authors declare that they have no competing interests.

## Authors' contributions

LMR: drafted and revised the manuscript, performed the statistical analyses and contributed to the interpretation of data. EMS: contributed to the conception and design, acquisition of data, interpretation of data, drafted and revised the manuscript. MP: contributed to the design of the experiments, interpretation of data, and revised the manuscript. THH: contributed to the conception and interpretation of the antibody laboratory results. EH: performed and interpreted laboratory analyses, drafted and revised the manuscript. LPT: contributed to the interpretation of the molecular pathology and biology aspects, drafted and revised the manuscript. All authors read and approved the final manuscript.

## Authors' information

No additional information.
